# Emerging roles of amphiregulin in disease pathogenesis and therapeutic promise

**DOI:** 10.1016/j.isci.2026.116499

**Published:** 2026-06-23

**Authors:** Nana Li, Junyi He, Meilin Liu, Lingfeng Zha

**Affiliations:** 1 Department of Cardiology, Union Hospital, Tongji Medical College, Huazhong University of Science and Technology, Wuhan 430022, China; 2 Hubei Key Laboratory of Biological Targeted Therapy, Union Hospital, Tongji Medical College, Huazhong University of Science and Technology, Wuhan 430022, China; 3 Hubei Provincial Engineering Research Center of Immunological Diagnosis and Therapy for Cardiovascular Diseases, Union Hospital, Tongji Medical College, Huazhong University of Science and Technology, Wuhan 430022, China

**Keywords:** Disease, Molecular biology

## Abstract

Amphiregulin (AREG) is an EGF-like ligand that binds EGFR to activate signaling pathways governing cellular proliferation, differentiation, tissue repair, and immune responses. This review integrates AREG’s structural characteristics and signaling mechanisms with its biological functions derived from cell-based and animal models. We critically evaluate AREG’s mechanistic involvement in cardiovascular, respiratory, intestinal, neoplastic, and infectious diseases, highlighting its dual roles in tissue protection and pathogenesis. The broader significance lies in discussing AREG’s translational potential and associated therapeutic challenges for these diseases.

## Introduction

Amphiregulin (AREG) is a unique single-chain hyper hydrophilic glycoprotein. AREG, which was initially found to suppress proliferation of some tumor cell lines and also induced the proliferation of normal cells such as fibroblasts and keratinocytes, is a bi-functional growth factor named amphiregulin.^1^ AREG was first isolated in 1988 by Shoyab from a conditioned medium of human breast cancer cell line MCF-7 cells.^1^ AREG, a member of the epidermal growth factor (EGF) family, is widely expressed in a variety of tissues and organs, and is closely related to the EGF receptor (EGFR) in a paracrine, autocrine, or paracrine manner. When binding to EGFR, it triggers intracellular signaling cascade, playing a key role in maintaining tissue homeostasis, promoting damage repair and regulating immune inflammation.^2^^,^^3^ In different microenvironments, hormones (such as luteinizing hormone and prostaglandin E2) and cytokines (such as IL-33 and IL-3) induce different types of activated immune cells (including innate lymphoid cell type 2 [ILC2], basophils, eosinophils, macrophages, mast cells, dendritic cells, neutrophils, CD8^+^ T cells, CD4^+^ T cells, and regulatory T cells [Tregs]) secret AREG. AREG takes part in various important pathophysiological processes, including autophagy, apoptosis, angiogenesis, regulation of immunity, and plays a vital role in tissue damage repair. AREG plays different biological roles under physiological and pathological conditions, for example, AREG is involved in the development and maturation of female oocytes and mammary glands.^4^^,^^5^ During acute or chronic inflammation, AREG promotes the restoration of tissue integrity and induces immune tolerance.^6^^,^^7^ Unlike other EGFR ligands such as EGF or tumor growth factor-α (TGF-α), AREG exhibits lower affinity for EGFR, fails to induce rapid receptor internalization, and can persist on the cell surface, leading to sustained signaling that favors differentiation over proliferation.^8^^,^^9^ Furthermore, AREG is uniquely produced by immune cells (e.g., ILC2s and Tregs) in response to inflammatory stimuli, underscoring its specialized role in immune modulation and tissue repair beyond general EGFR biology.^2^^,^^10^^,^^11^ Throughout this review, we highlight these distinctive features wherever relevant. In this review, the structure, signaling pathway and biological function of AREG under physiological and pathological conditions will be reviewed based on the recent research progress.

## Structure of AREG

**Figure 1:**
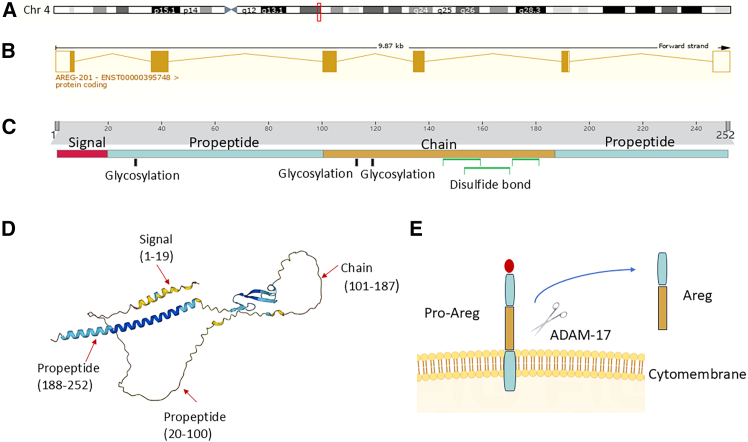
AREG encodes a transmembrane precursor that is cleaved by ADAM‑17 to release a soluble EGF‑like ligand (A) AREG genomic DNA spans approximately 10 kb. (B) Transcription generates a 1.4 kb mRNA that encodes pro‑AREG. (C) Pro-AREG is a transmembrane polarized glycoprotein precursor. (D) Pro-AREG consists of 252 amino acids. (E) ADAM-17 cleaves pro-AREG at the plasma membrane, releasing a soluble 84-amino acid AREG protein. Mature soluble AREG comprises an N-terminal N-glycosylated heparin-binding hydrophilic domain and a C-terminal EGF-like domain

The total length of AREG genomic DNA is about 10 kb, located in the human gene 4q13-21 region. During transcription, a 1.4 kb length AREG mRNA is generated, which encodes a transmembrane polarized glycoprotein precursor (pro-AREG) containing 252 amino acids^3^ (Figure 1). ADAM-17, a member of the disintegrin and metalloproteinase (ADAM) family, cleaved the extracellular functional domain of pro-AREG protein in the plasma membrane of the cell, releasing a soluble AREG protein containing 84 amino acids^12^ (Figure 1). Mature soluble AREG proteins contain the hydrophilic amino terminal of the N-glycosylated heparin-binding domain and the carboxyl terminal of the EGF-like domain. The bioactive domain of AREG consists of six essential cysteine 26 residues spaced CX7CX4CX10CX1CX8C, where C stands for cysteine and X can be any amino acid.^13^ AREG was classified as a member of the EGFR ligand family because of its significant homology with other members of the EGF family, such as 38% homology with EGF and 32% homology with TGF-α, and its ability to competitively bind to EGFR and induce autocrine or paracrine activation of EGFR.^13^^,^^14^ The researchers demonstrated that AREG can induce EGFR phosphorylation in different cancer cell lines.^13^

## Expression regulation of AREG

The expression activity of AREG is regulated by transcriptional level and post-transcriptional level.^3^^,^^4^ Several studies have reported transcriptional activators of AREG, it includes hypoxia-inducible factor 2α (HIF2α),^15^ cAMP response element (CRE),^16^ SP1 binding consistent element,^3^ Wilms tumor suppressor factor WT1 response element (WRE),^17^ β-catenin,^18^ ETS/ATF binding site (EBS),^3^ and serum reactor SRE.^3^ Estrogen receptor binding element (ERE)^19^ and AP1^20^ can also stimulate the expression of AREG. The tumor suppressor genes BRCA-1 and IKKalpha are transcriptional suppressors of AREG, which inhibit the expression of AREG in breast cells and keratinocytes, respectively.^21^^,^^22^ At the post-transcriptional level, the EGFR ligand, human antigen R (HuR), regulates AREG expression by increasing the stability of AREG mRNA.^23^^,^^24^ In addition, AREG or other EGF ligands can activate EGFR through a self-feedback mechanism, thereby inducing AREG transcription.^25^^,^^26^ Some hormones (such as luteinizing hormone,^27^ prostaglandin E2,^28^ and parathyroid hormone^29^) and growth factors (such as insulin-like growth factor-1 [IGF-1]^30^ and transforming growth factor-β [TGF-β]^31^) can also effectively induce AREG expression. Cytokine can effectively stimulate the expression of AREG in immune cells. For example, IL-33 can stimulate the expression of AREG in congenital lymphoid cells, and IL-3 significantly stimulates the expression of AREG in basophil cells.^6^^,^^7^ In addition, hypoxia and smoking can also induce AREG expression.^32^^,^^33^

**Figure 2:**
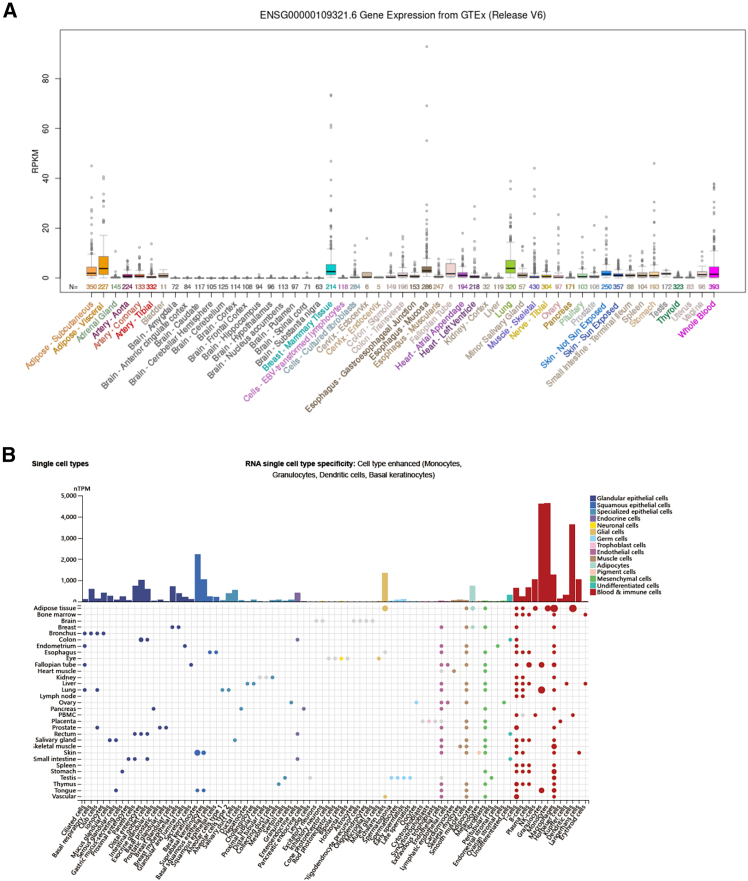
AREG expression can be detected in a variety of tissues (A) Data were derived from the public database GTEx portal (https://gtexportal.org). (B) Data were obtained from the public database the human protein atlas (https://www.proteinatlas.org).

AREG, a member of the EGF family, is expressed by a variety of epithelial cells and mesenchymal cells during development and under homeostasis,^3^ AREG expression can be detected in a variety of tissues (Figure 2). AREG plays an important role in regulating lung morphogenesis, keratinocyte proliferation, and mammary gland development.^2^ Mice with AREG gene defects have no obvious abnormalities under normal conditions, but when tissue is damaged or inflammation occurs, the repair ability is reduced.^34^ AREG is rapidly expressed in pathological conditions, such as when keratinocytes are injured.^35^ AREG is also expressed by many activated immune cells under different inflammatory stimuli, including ILC2,^36^ basophils,^37^ eosinophils,^38^ macrophages,^39^ mast cells,^10^ dendritic cells,^40^ neutrophils,^41^ CD8^+^ T cells,^42^ CD4^+^ T cells,^11^ and Treg.^6^^,^^43^

## Signaling pathway of AREG activation

After transcription, translation, protein processing and transportation, and proteolytic treatment, AREG proteins function in EGFR dependent and EGFR independent ways. AREG signaling pathway can be triggered by diverse ways such as kernel translocation, paracrine, juxtacrine, autocrine, and exosome inclusion. It is reported that a single exosome contains an average of 24 AREG molecules, which are quickly internalized by the recipient cell and are five times more effective at promoting cell invasion than the equivalent amount of recombinant AREG.^44^

When AREG binds to EGFR in a competitive manner, it can activate an intracellular multikinase phosphorylation cascade.^3^ *In vitro* experiments, low doses of AREG (3 and 10 nM) bind to EGFR monomer and induce receptor dimerization, while higher doses of AREG (30, 100, and 300 nM) bind primarily to pre-existing receptor dimers.^45^ In addition, AREG, when combined with EGFR, induces the activation of ErbB2, ErbB3, and ErbB4, other members of the EGF receptor family.^46^ The downstream signaling pathways related to AREG/EGFR activation include RAS/RAF/MEK/ERK, PI3K/AKT, JNK, p38, mTOR, PLC-γ/PKC, STAT, etc.^2^^,^^3^^,^^31^ These pathways are shared among all EGFR ligands; however, the duration, intensity, and biological outcome of signaling differ. Low-affinity binding of AREG fails to induce rapid EGFR internalization.^3^^,^^8^^,^^9^ Subsequently, activation of the signaling pathway further leads to the activation of related transcription factors, regulating the expression of downstream AREG-related target genes, thereby triggering various cellular responses, such as proliferation, motility, survival, invasion, and angiogenesis.^3^^,^^27^

It has been reported that AREG of nuclear translocation functions in a manner independent of EGFR. AREG precursors translocation from the plasma membrane to the nuclear membrane via retrograde transport, interacting with laminin type A and downregulating global transcription.^27^ In the DNA damage response, nuclear AREG regulates microRNA processing by binding to DDX5 and Drosha and regulates apoptosis.^47^ In addition, nuclear localization of AREG was also observed in syncytiotrophoblast cells of placental villi and ovarian surface epithelial cells.^27^ There are still many unknowns about the role of nuclear translocation AREG, which need to be further explored.

## Biological function of AREG

**Figure 3:**
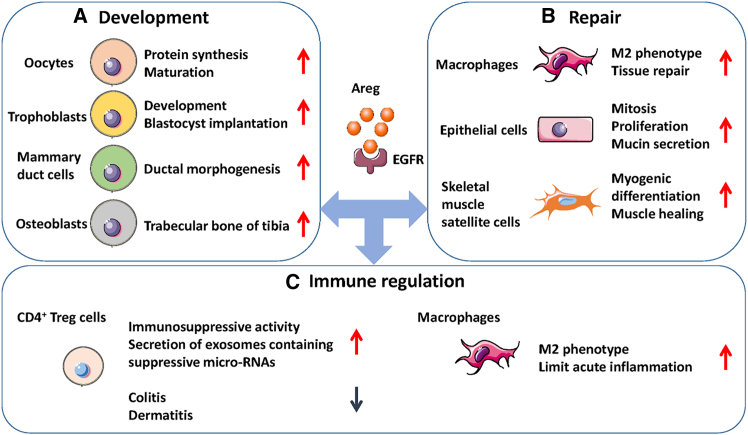
AREG protein binds EGFR, regulating diverse cellular processes, promoting growth, development, damage repair, and modulating immune responses

AREG protein binds to EGFR, regulates various cellular processes, such as immune cells and parenchymal cells, participates in growth and development, promotes damage repair, and coordinates immune response (Figure 3). It plays different biological roles under physiological and pathological conditions.^3^

### Participate in growth and development

AREG expression can be detected in many normal tissues, such as the spleen, lungs, ovaries, colon, bone marrow, testicles, blood, pancreas, kidney, placenta, and mammary gland.^48^ AREG protein is also found in the serum and colostrum of healthy people.^49^^,^^50^

The current study shows that EGFR signaling regulates a key part of the female reproductive system. For example, luteinizing hormone stimulates the secretion of AREG, and AREG secreted paracellular in the cumulus is involved in driving the synthesis of specific proteins in oocytes and is closely related to the maturation of oocytes and their development into embryos.^51^^,^^52^ In early pregnancy, progesterone induces AREG expression in the uterine epithelium, promoting trophoblast growth and blastocyst implantation.^3^^,^^53^ Estrogen receptor α regulates mammary duct development through AREG, and the pubertal mammary duct development of AREG knockout mice is significantly inhibited.^3^^,^^34^ In addition to regulating the development of the female reproductive system, AREG is also involved in regulating the branching and morphogenesis of the prostate, lung, kidney, etc.^3^ AREG knockout mice showed atrophy of gastric acid glands and fundus mucosal lesions.^54^ During acute and chronic injury, TLR-4 induces AREG production and promotes the repair and protection of intestinal tissue.^55^^,^^56^ Although AREG was under-expressed in healthy livers, the AREG knockout mice showed hepatocyte damage.^57^ The expression level of AREG in liver is significantly increased during liver regeneration and acute liver injury, which promotes the regeneration and protection of liver cells.^57^^,^^58^ AREG is also involved in the physiological development of bone. AREG expression is regulated by parathyroid hormone and other osteo stimulating hormones in bone tissue and osteoblasts. The study found that the tibial trabecular bones of AREG knockout mice were significantly reduced compared to wild-type mice.^29^ In addition, AREG is a key immune response cytokine produced by T cells.^11^

### Promote tissue repair

It was found that after tissue injury, adenosine induced up-regulation of IL-33, promoted ILC2 expression of AREG, and regulated macrophage differentiation into M2 type.^59^^,^^60^ By expressing mitogen IGF-1 and arginase, M2 macrophages play an important role in promoting tissue repair and limiting acute inflammation.^60^ IGF-1 works synergistically with members of the EGF family to enhance EGFR signaling and promote epithelial cell proliferation.^61^ In keratinocytes, IGF-1 promotes AREG expression and positive feedback promotes cell proliferation.^30^ This evidence suggests that AREG and IGF-1 work together to stimulate epithelial cell mitosis and promote tissue repair after tissue damage caused by infection. AREG also activates human tenocytes as dendritic cell-derived AREG binds to tenocyte EGFR and promotes proliferation, especially in diseased tenocytes, suggesting a role in tendon repair.^62^

Unlike other EGFs, AREG can induce not only mitosis, but also cell differentiation. High affinity binding of other EGF ligands to EGFR induces rapid internalization and degradation of EGFR, leading to activation and termination of downstream signaling pathways, inducing cell proliferation.^8^ However, the low affinity binding of AREG to EGFR fails to induce receptor internalization, resulting in continuous downstream signaling and inducing cell differentiation.^9^^,^^63^ Differences in signal transduction determine the dynamics of gene expression. After muscle injury, Treg accumulates at the site of muscle injury and promotes *in vitro* myogenic differentiation of skeletal muscle satellite cells through the secretion of AREG. *In vivo*, muscle healing can be promoted by injecting recombinant AREG protein into injured muscles.^6^ *In vitro*, AREG induces differentiation of neuronal PC12 cells.^64^ The differentiation of mammary epithelial cells, nephrogenic MDCK cells and myoepithelial cells was also induced by AREG.^65^

### Regulation of immune inflammation

Effective removal of local inflammation is the basis of wound healing.^66^ Mast cells and Tregs are key cells in immunosuppression.^67^ Mast cells secrete IL-10 to regulate local immune responses.^68^ In a mouse model of colitis, Tregs did not inhibit the development of colitis when mast cell-derived AREG was absent. In mouse tumor inoculation models, Tregs were unable to induce immunosuppression when mast cell-derived AREG was absent, nor were they effective in inhibiting skin inflammation. Adoptive transfer of mast cells from the bone marrow of wild-type mice inhibited skin inflammation, while adoptive transfer of mast cells isolated from the bone marrow of mice with mast cell-derived AREG deficiency did not inhibit skin inflammation. The above study revealed that AREG produced by mast cells can enhance the local immunosuppressive effect of Treg.^10^ Some studies have found that AREG can promote the secretion of exosomes containing immunosuppressive microRNAs by Treg cells, act on effector T cells, and inhibit local immune inflammation.^69^ In skin allergies, basophil derived AREG plays an importance role in enhancing the immunosuppressive function of Tregs.^70^ In addition to mast cells and eosinophils, Tregs themselves also express AREG, which may have a positive autocrine feedback loop.^6^ However, it is unclear to what extent AREG secreted by Treg cells directly promotes wound healing and indirectly contributes to the resolution of local inflammation. In a mouse liver cancer model, AREG secreted by HCC cells binds to EGFR and enhances Treg activity through mTOR signaling.^4^ In summary, several studies have revealed the ability of AREG to enhance Treg’s ability to inhibit local immune response and induce peripheral tolerance.

AREG can exert both pro-repair and pro-inflammatory effects depending on cellular source, microenvironment, and timing. For example, Treg-derived AREG promotes muscle repair^6^ and lung repair after influenza,^71^ whereas mast cell-derived AREG enhances immunosuppression without direct inflammation.^10^ In chronic infection or cancer, AREG from tumor cells or macrophages augments Treg-mediated suppression, perpetuating inflammation and disease progression.^72^^,^^73^ Thus, the net outcome is highly context-dependent. Future studies should dissect the molecular switches that determine whether AREG promotes resolution or persistence of inflammation.

## AREG is involved in disease

To provide a balanced and structured synthesis, we organize the following disease sections around three common axes: the cellular source of AREG, the target cell type and downstream signaling pathway, and the functional outcome (protective, pathogenic, or mixed). Because AREG takes part in various important biological processes, it plays an important role in the pathophysiological processes of the human body, and its abnormal regulation can cause diseases of multiple systems, such as cardiovascular diseases, respiratory diseases, intestine diseases, tumors, and infectious diseases. More and more studies have found that AREG is involved in the occurrence and development of these diseases. For a structured overview, Table 1 summarizes the dominant cellular sources, target cells, functional outcomes, and evidence strength of AREG across major disease contexts.

**Table 1:** Summary of the dominant cellular sources, target cells, functional outcome, and strength of evidence for AREG in key disease contexts

Disease/organ context	Dominant producing cells	Target cells	Functional outcome	Strength of evidence	Ref
Myocardial infarction (post-MI)	cardiac macrophages, Tregs	cardiomyocytes	protective (anti-apoptotic, autophagy regulation)	strong (mouse), limited human	Sugita et al.,^74^ Fujiu et al.,^75^ Li et al.,^76^
Atherosclerosis (coronary plaque)	αβ T cells	smooth muscle cells, fibroblasts	pathogenic (proliferation, fibrosis)	moderate (human plaque)	Chowdhury et al.,^77^
Pulmonary hypertension	endothelial cells	endothelial cells, immune cells	protective (loss of AREG is pathogenic)	moderate (mouse & human)	Florentin et al.,^78^
Lung (influenza infection)	Tregs	epithelial cells, mesenchymal cells	protective (tissue repair)	strong (mouse)	Kaiser et al.,^71^ Arpaia et al.,^79^
Lung (asthma)	mast cells, epithelial cells	airway epithelium, smooth muscle	pathogenic (mucus metaplasia, remodeling)	moderate (human/mouse)	Okumura et al.,^80^ Enomoto et al.,^81^
Intestine (colitis/radiation)	ILC2s, epithelial cells	intestinal epithelium	protective (regeneration, barrier repair)	strong (mouse)	Chen et al.,^82^ Monticelli et al.,^83^
Crohn’s disease (intestinal fibrosis)	Tregs, myofibroblasts, Th17 cells	fibroblasts	pathogenic (fibrosis, stricture)	moderate (human/mouse)	Wang et al.,^84^ Zhao et al.,^85^ Wang et al.,^86^
Colorectal cancer	tumor cells, Tregs	epithelial cells, T cells	mixed (proliferation, immune suppression)	strong (mouse), moderate human	Guernsey-Biddle et al.,^87^
Hepatocellular carcinoma	tumor cells, macrophages	hepatocytes, Tregs	pathogenic (proliferation, immune evasion)	moderate (human/mouse)	Castillo et al.,^88^ Pardo-Saganta et al.,^89^ Castillo et al.,^90^
Allogeneic heart transplantation	Tregs	fibroblasts, smooth muscle	pathogenic (chronic rejection)	strong (mouse)	Warunek et al.,^91^
Acute graft-versus-host disease (GVHD)	not specified (biomarker)	–	prognostic (high AREG → poor outcome)	human (clinical trials)	Holtan et al.,^92^ Zeka et al.,^93^

### AREG and cardiovascular diseases

#### Areg in cardiac diseases

**Figure 4:**
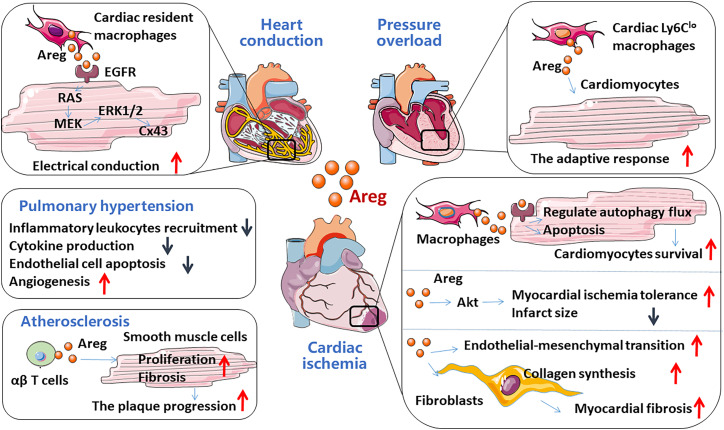
AREG contributes to cardiovascular disease pathogenesis

AREG is involved in the progression of cardiovascular disease (Figure 4). AREG can interact with EGFR, which is widely expressed in cardiomyocytes, and initiates downstream signaling pathways. AREG secreted by cardiac macrophages can regulate the phosphorylation and translocation of connexin 43 in cardiomyocytes, strengthen the communication between cardiomyocytes, maintain the normal conduction of cardiac impulses, and thus prevent sudden death during cardiac stress. If AREG in macrophages is knocked out, it leads to disruption of the gap connections, which can lead to fatal arrhythmias during acute stress.^74^ In the pressure overload model, AREG is an important cardioprotective mediator produced by cardiac Ly6C^lo^ macrophages, promoting cardiac resilience to pressure overload by inducing a hypertrophic response of cardiomyocytes.^75^ In the heart of heart failure, CD8^+^ T cells play a key regulatory role in the transformation of cardiac resident macrophages and infiltrating macrophages into heart-protective macrophages expressing AREG, Igf1, and other growth factor genes, and are crucial for the myocardial adaptive response after pressure overload.^94^ In addition, during myocardial ischemia, recombinant AREG protein treatment can activate the survival kinase Akt, increase myocardial ischemia tolerance, and alleviate myocardial ischemia reperfusion injury in mice.^95^ In the myocardial infarction model, AREG expression was elevated at the infarct junction after MI, and AREG deficiency aggravated poor ventricular remodeling after MI. AREG^−/−^ mice showed worsening cardiac function, decreased survival, and increased infarct size and interstitial fibrosis after MI. Further mechanism revealed that AREG inhibited autophagosome over synthesis by activating PI3K/Akt/mTOR pathway, while upregulated lysosome acidity promoted autophagosome clearance and inhibited cardiomyocyte apoptosis.^76^ AREG inhibits doxorubicin induced cardiomyocyte apoptosis.^96^ In addition, paracrine AREG of Treg enhances cardiomyocyte proliferation and regulates the regeneration of newborn hearts.^97^ The previous studies have shown the protective effect of AREG in cardiovascular diseases.

It has been reported that AREG promotes myocardial hyper fibrosis by increasing collagen synthesis in myocardial fibroblasts and inducing endothelial mesenchymal transformation after MI. It has also been found that AREG knockout mice have increased infarct size after myocardial ischemia, and recombinant AREG protein preconditioning can increase myocardial ischemia tolerance and reduce myocardial infarction size.^95^ The reported different effects of AREG after MI may be related to the different effects of AREG on fibroblasts and cardiomyocytes. By developing nanomedicine with specific affinity to cardiomyocytes, it is beneficial to mitigate the adverse effects of AREG on fibroblasts.^98^^,^^99^ As mentioned earlier, AREG promotes tissue repair. After muscle injury, the accumulated Treg at the site of muscle injury promotes *in vitro* myogenic differentiation of skeletal muscle satellite cells through the secretion of AREG, and promotes muscle healing.^6^ A key link in wound repair is the deposition of connective tissue proteins such as collagen and fibronectin in and around the open wound. In the injured tissue, myofibroblasts secrete collagen to promote wound contraction and stability, thus achieving effective healing. However, during long-term chronic inflammation, excessive activation and differentiation of myofibroblasts and excessive deposition of collagen can trigger organ fibrosis and lead to impaired organ function.^100^ Because cardiomyocyte apoptosis mainly occurs in the early post-MI period, excessive fibrosis in the late MI period may impair cardiac function.^99^^,^^101^^,^^102^ In future studies, by adjusting the time point of administration reasonably, the adverse effects of AREG on cardiomyocyte autophagy and apoptosis could be minimized while retaining the regulatory effect of AREG on cardiomyocytes.

#### Areg in vascular pathology and atherosclerosis

Similar to its time-dependent effects in post-myocardial infarction, AREG also displays opposing roles in vascular diseases depending on the pathological context. AREG and its receptor EGFR are downregulated in patients with pulmonary hypertension. Both *in vivo* and *in vitro* studies have indicated that the deficiency of AREG and EGFR in endothelial cells increases inflammatory leukocyte recruitment, cytokine production, and endothelial cell apoptosis, while reducing angiogenesis. Consequently, AREG and EGFR deficiency promote pulmonary endothelial cell death, immune cell infiltration and vascular remodeling, leading to pulmonary hypertension.^78^ Studies also have found that AREG promotes the progression of cardiovascular disease. In coronary plaque, there is a population of αβ T cells with high AREG expression, which promotes plaque progression by inducing smooth muscle cell proliferation and fibrosis.^77^ Thus, the net outcome of AREG signaling in the vasculature is highly context-specific—protective when its expression is maintained in pulmonary endothelium, yet detrimental when overproduced in atherosclerotic plaques—highlighting the need for precise spatial and temporal modulation in future therapies.

### AREG and respiratory diseases

AREG is expressed in airway epithelial cells and induced in immune cells upon injury.^71^^,^^103^ Treg-derived AREG is involved in maintaining lung function during influenza virus infection. Mice with AREG knocked out in Treg had significantly lower blood oxygen saturation.^79^ The mechanism study found that AREG secreted by Treg promoted the survival of Col14a^+^ mesenchymal cells in the highly inflammatory tissue environment after influenza virus exposure, and increased expression of IL-33 in Col14a^+^ mesenchymal cells further stimulated the secretion of AREG. This positive feedback loop further strengthened the interaction between Treg cells and stromal cells and promoted the transition of lung Treg cells to a state that promotes tissue repair.^71^ It has also been reported that lipopolysaccharide in the bacterial outer membrane causes damage to lung tissue and alveolar cells after Gram-negative bacteria infection. AREG is a protective factor of alveolar epithelial cells by preventing TNF-α-induced apoptosis through EGFR.^104^ By activating the EGFR-AKT pathway, AREG effectively reduces the flow of protein-rich fluid into the alveolar space, inhibits the infiltration of inflammatory cells, downregulates the expression of inflammatory mediators, and ultimately protects lung tissue from lipopolysaccharides-induced acute lung injury by strengthening the epithelial barrier.^105^ Similarly, this protective role is also observed in other pulmonary vascular diseases. In pulmonary hypertension, AREG plays a protective role by suppressing endothelial cell apoptosis, inflammation, and vascular remodeling.^78^ However, the function of AREG is not always protective. In acute asthma, amphiregulin promotes airway epithelial cell proliferation and mucous cell metaplasia, potentially contributing to airway remodeling.^81^ Thus, the role of AREG in lung diseases is highly context-dependent.

### AREG and intestine diseases

In the intestine, AREG exhibits a dual, context-dependent role. Under homeostatic or acute injury conditions, AREG exerts protective effects by promoting epithelial barrier repair and limiting inflammation.^82^ Specifically, intestinal epithelial cell-derived AREG, induced by neutrophil-derived TGFβ, restores barrier function and ameliorates colitis, while IL-33-activated ILC2s produce large amounts of AREG that facilitate epithelial regeneration and tissue protection.^83^ Conversely, in chronic inflammatory and neoplastic settings, AREG becomes a pathogenic mediator. In Crohn’s disease (CD), AREG derived from regulatory T cells, intestinal myofibroblasts, and Th17 cells drives intestinal fibrosis by activating mTOR, MEK, and PI3K/AKT signaling pathways, promoting extracellular matrix deposition and stricture formation.^84^^,^^85^^,^^86^ Furthermore, in colorectal cancer, AREG is frequently overexpressed and contributes to tumor progression, while also serving as a predictive biomarker for response and resistance to EGFR-targeted therapies such as cetuximab and panitumumab.^87^ Thus, AREG acts as a protective factor in acute injury but exacerbates fibrosis and tumorigenesis in chronic intestinal diseases.

### AREG and tumors

**Figure 5:**
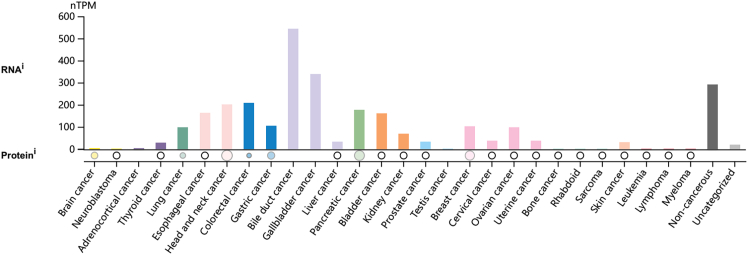
AREG expression is upregulated in many tumors

AREG takes part in the occurrence and development of tumors. AREG expression is upregulated in many tumors, such as lung, skin, bladder, liver, ovarian, stomach, prostate, breast, head and neck cancers, biliary tract cancers, colon cancers, and pancreatic cancers^3^ (Figure 5). From the perspective of molecular function mechanism, AREG participates in the occurrence and development of cancer as a cancer promoting factor.^106^ In mice, overexpression of AREG induces abnormal development and proliferation of pancreatic duct cells. AREG is also highly expressed in cirrhotic liver, suggesting that AREG may be related to the development of liver cancer.^88^^,^^107^ Overexpression of AREG promotes the growth of breast, liver, colon, lung, and pancreatic cancer cells and promotes the development of cancer.^3^ The study found higher AREG protein levels were associated with advanced ovarian cancer, and patients with high AREG expression in their tumors had obviously shorter survival than patients with low AREG expression.^108^

Activated monocyte-derived dendritic cells secrete AREG upon stimulation with ATP or oxidized phospholipids, thereby promoting lung cancer progression.^40^^,^^109^ AREG promotes the progression of TLR-4-associated inflammatory colorectal cancer.^110^ AREG is associated with invasion and migration of various tumor cells.^2^^,^^111^ The high expression of AREG in colorectal cancer tissues is a predictor of liver metastasis,^112^ and exosomes containing AREG significantly enhance the invasion potential of breast cancer and colon cancer cells.^44^ Liver injury and hepatitis induce AREG expression,^89^ and the autocrine feedback mechanism of AREG promotes the maintenance of tumor phenotype of hepatocellular carcinoma cells.^90^ It has also been shown that AREG enhances the mitogenic effect of other growth factors such as fibroblast growth factor 19 (FGF19) and triggers the expression of tumorigenic factors and matrix metalloproteinases in liver cancer cells.^3^^,^^113^

In addition to act directly on cancer cells, AREG also promotes cancer progression by inducing tumor immune tolerance. AREG is secreted by tumor-associated mast cells, which can enhance the inhibitory capacity of Treg and promote tumor immunosuppression after binding with the EGFR of Treg.^10^ In AREG knockout mice, immunizing B16 tumor-bearing mice with dendritic cells containing the immunogenic tumor antigen TRP2 peptide induced CD8^+^ T cell response and tumor rejection.^10^ In a mouse model infected with hepatitis B virus, increased liver AREG expression is strongly related to the presence of super-strong immunosuppressive EGFR^+^ CD4^+^ Treg cells, limiting the function of antiviral CD8^+^ T cells.^72^

Several studies have reported an association between AREG expression and chemotherapy resistance in certain cancer types, particularly hepatocellular carcinoma treated with sorafenib.^112^^,^^114^ Studies have also found that high expression of AREG is associated with resistance to EGFR-targeted therapy in gastric and breast cancer cell lines, as well as non-small cell lung cancer cells and patients.^3^ However, causality has not been firmly established, and the relationship appears context-dependent, likely involving multifactorial mechanisms beyond AREG/EGFR signaling alone. Further research is needed to validate these findings and to explore the potential of targeting AREG in overcoming drug resistance.

### AREG and infectious diseases

Mounting evidence indicated that AREG takes part in viral, bacterial, and parasitic infections.^115^ During influenza virus infection, Treg-derived Areg promotes mesenchymal cell survival and drives Tregs into a tissue-repair phenotype, thereby preserving lung function.^71^ Different from influenza virus infection, after infection with carcinogenic viruses such as HPV virus, AREG is an important autocrine factor promoting the proliferation of HPV immortalized cervical cells.^116^ Recombinant AREG promotes the growth of cervical cells, and the proliferation of breast and colon cancer cell lines depends on autocrine AREG.^115^ AREG expression was up-regulated in the liver of HBV-infected mice.^72^ It was found that AREG is involved in CD206^+^ macrophage-mediated immunomodulatory process, and M2 polarization related signaling pathway may promote the expression of AREG in macrophages.^73^ As previously mentioned, AREG enhances the suppression of local immune response by Treg, and the immunosuppressive activity of EGFR^+^ Treg in the liver of HBV-infected mice is greater than that of EGFR^−^ Treg. In the mouse HBV model, CD206^+^ macrophages in the liver produce AREG, and the expression of EGFR on the surface of Treg cells is upregulated. AREG promotes the activation of Treg cells and ultimately inhibits the anti-viral activity of CD8^+^ T cells.^72^^,^^73^ In the future, more researches are needed to fully understand the roles of AREG and Treg in HBV infection.

AREG also plays a key role in bacterial infections, and the peptides produced by AREG have antibacterial effects against some pathogens *in vitro*.^117^ *Neisseria gonorrhoeae* infection induces increased transcription of AREG. At the plasma membrane, the proteolytic cleavage pattern of AREG changes and a large amount of induced AREG is released into the cellular supernatant. This secreted AREG colocalizes with the bacteria attached to the plasma membrane, and *Neisseria gonorrhoeae* adheres to and subsequently invades the host cell.^118^ After *E. coli* infection, neonatal monocytes from cord blood had 11 times higher levels of soluble AREG than monocytes from adult donor peripheral blood.^119^ AREG may inhibit phagocytic induced cell death after bacterial infection and hinder the effective resolution of inflammatory response.^72^

Parasite invasion and subsequent inflammatory response can lead to damage in the upper cortex, and local epithelial cell proliferation has a protective effect by promoting healing of damaged epithelial cells and parasite excretion.^120^ The growth and division of colon epithelial cells were reduced and the elimination time of trichomonas was prolonged after infection with the intestinal parasite Trichomonas in AREG knockout mice, suggesting that AREG plays a key role in promoting the protective epithelial response of intestinal tissue. In wild-type mice infected with parasites, proliferation of the epithelial cell layer was strongly associated with CD4^+^ T cells, suggesting the significance of the interaction between immune cells and epithelial cells in regulating AREG protection.^2^ Other studies have reported that AREG expression by Th2 cells induces epithelial cell proliferation and plays a pivotal role in the primary defense of nematodes.^2^^,^^11^ Wild-type and AREG knockout mice showed similar IL-4, IL-5, and IL-13 responses after infection with murine T. Gondii, but AREG knockout mice showed reduced CD4^+^ T cells mediated epithelial cell proliferation and migration, and showed a halt in toxoplasma clearance.^11^ Adoptive transfer of AREG-expressing bone marrow cells significantly improved the parasite removal ability of AREG knockout mice.^11^ In mice infected with Strongylus brazilianus, AREG expression promotes the process of tissue recovery after lung injury.^11^ In sero-positive but asymptomatic people with visceral leishmaniasis, there is a good correlation between Treg expression and the amount of AREG, with elevated plasma AREG levels and increased antigen-specific AREG release in whole blood.^121^ Table 2 groups infectious pathogens based on whether AREG exerts a host-protective or pathogen-exploitative role and highlights the underlying mechanistic themes.

**Table 2:** Categorization of infectious pathogens based on whether AREG plays a host-protective role or is exploited by the pathogen

Pathogen/disease	Host-protective role	Pathogen-exploitative role	Key mechanisms	Ref
Influenza virus	yes (Treg-derived AREG promotes lung repair)	no	Treg-mesenchymal cell positive feedback (IL-33)	Kaiser et al.,^71^ Arpaia et al.,^79^
Hepatitis B virus (HBV)	no	yes (suppresses CD8^+^ T cell immunity)	AREG from CD206^+^ macrophages upregulates EGFR on Tregs → enhances Treg suppression	Dai et al.,^72^ Dai et al.,^73^
Human papillomavirus (HPV)	no	yes (autocrine growth factor)	AREG drives proliferation of HPV-immortalized cervical cells	Woodworth et al.,^116^
*Neisseria gonorrhoeae*	no	yes (altered AREG processing, bacterial adherence)	infection changes AREG proteolytic cleavage; secreted AREG colocalizes with adherent bacteria	Löfmark et al.,^118^
*Escherichia coli* (neonatal)	unclear	possibly (inhibits phagocytic cell death)	higher soluble AREG in neonatal monocytes; may hinder resolution of inflammation	Platen et al.,^119^
Helminths (e.g., Trichuris, Toxoplasma, Strongylus)	yes (Th2/ILC2-derived AREG)	no	AREG drives epithelial proliferation, parasite expulsion, and tissue repair after lung injury	Zaiss et al.,^2^, Zaiss et al.,^11^
*Leishmania donovani* (asymptomatic)	correlative (AREG elevated)	not defined	elevated plasma AREG and antigen-specific release in whole blood	Singh et al.^121^

### AREG and transplant biology

Emerging evidence suggests that AREG plays a complex and context-dependent role in transplantation. In a murine heart transplantation model, graft fibroblast-derived IL-33 potently induced AREG expression in recipient Tregs; unexpectedly, Treg-derived AREG exacerbated chronic rejection by promoting fibroblast proliferation, fibrosis and graft vasculopathy, and hearts from recipients with AREG-deficient Tregs showed markedly less fibrosis and vasculopathy.^91^ In small-for-size liver transplantation, AREG administration stimulated liver regeneration, improved liver function, and increased survival through EGFR-dependent pathways, while AREG neutralizing antibody blunted regeneration.^58^ Moreover, circulating AREG levels measured during two prospective clinical trials (NCT02525029 and NCT02953678) were identified as a monitoring biomarker for life-threatening acute graft-versus-host disease, with high AREG associated with poor steroid response and lower survival.^92^ In pediatric intestinal biopsies, AREG expression scores were higher in patients with severe graft-versus-host disease (GvHD) and inflammatory bowel disease (IBD) compared to controls and patients with mild or no GvHD, suggesting that AREG staining may serve as an additional marker for severe inflammation in GvHD and IBD.^93^ Collectively, these findings position AREG as both a potential therapeutic target and a prognostic biomarker in transplantation, but its context-dependent duality necessitates careful patient stratification.

## Summary and prospect

Consensus exists that AREG is a key mediator of tissue repair and immune regulation across multiple organs. In the cardiovascular system, AREG from cardiac macrophages and Tregs exerts cardioprotective effects by reducing apoptosis and enhancing autophagy.^74^^,^^75^^,^^76^ In the lung, Treg-derived AREG promotes epithelial repair after influenza infection and protects against acute lung injury,^71^^,^^79^ while in the intestine AREG, supports epithelial regeneration after colitis or radiation damage.^82^^,^^83^ In cancer, AREG frequently acts as a pro-tumorigenic factor by promoting tumor cell proliferation, invasion, and Treg-mediated immune suppression.^10^^,^^88^

Despite these advances, several important questions remain unresolved. First, the dual protective versus pathogenic roles of AREG—for example, its beneficial effects in acute myocardial infarction versus its detrimental contributions to atherosclerosis and pulmonary hypertension—are not fully understood at the mechanistic level. Second, whether AREG acts as a damage-associated molecular pattern (DAMP) or strictly as a regulated growth factor requires further investigation. Third, the relative contribution of AREG versus other EGFR ligands (EGF, TGF-α, and HB-EGF) in most physiological and pathological settings remains unclear. Fourth, the translational potential of targeting AREG is hampered by a lack of cell-type-specific delivery systems and by the complexity of its context-dependent functions.

Given the pleiotropic actions of AREG, several therapeutic strategies are being explored, including neutralizing antibodies against AREG, EGFR tyrosine kinase inhibitors, and ligand traps. Predicted benefits include suppression of tumor growth in AREG-dependent cancers (e.g., colorectal and hepatocellular carcinoma), mitigation of fibrosis in chronic intestinal and pulmonary diseases, and enhancement of tissue repair after acute injury (e.g., myocardial infarction). However, major challenges must be overcome. Non-specific EGFR blockade risks interfering with homeostatic tissue repair, causing immunosuppression that increases susceptibility to infections, and inducing cardiovascular or dermatological toxicities. Furthermore, the protective role of AREG in certain contexts (e.g., cardiac protection and lung repair) suggests that global inhibition may be harmful. Future research should focus on developing context-specific and cell-type-targeted approaches, such as antibody-drug conjugates or nanoparticle-mediated delivery, to selectively modulate AREG activity in diseased tissues while preserving its homeostatic functions. A deeper understanding of the signaling nodes that distinguish AREG-specific effects from general EGFR signaling will also be essential for designing safer and more efficacious therapies.

In conclusion, AREG is a central regulator of tissue homeostasis, repair, and disease pathogenesis. Clarifying its context-dependent mechanisms and resolving the controversies highlighted previously will pave the way for harnessing AREG as a therapeutic target in cardiovascular, respiratory, intestinal, neoplastic, and infectious diseases.^122^^,^^123^

## Acknowledgments

We appreciate all participations and supports of this study. This work was supported by 10.13039/501100001809National Natural Science Foundation of China (grant no. 82200319 to L.Z.).

## Author contributions

All authors contributed to the study conception and design. Material and figure preparation were performed by N.L., L.Z., J.H. and M.L. The first draft of the manuscript was written by N.L. and L.Z. All authors commented on previous versions of the manuscript. All authors read and approved the final manuscript.

## Declaration of interests

The authors declare no competing interests.
